# Lancashire County Asylums

**Published:** 1902-01-04

**Authors:** 


### LANCASHIRE COUNTY ASYLUMS.

CHANGES in the medical staffs of the Lancashire Asylums have lately taken place. With the retirement of Mr. Rooke Ley from the Prestwich Asylum a vacancy occurred in the medical superintendency of that asylum, which was filled by transferring Mr. Perceval from Whittingham. At the latter Mr. Perceval had charge of something like two thousand lunatics, which ought to be enough to satisfy the longings of most men; but at Prestwich he will have an additional five hundred to look after. At present the salary is the same, but the possibilities are greater and the position in many ways is better.

Mr. Perceval's successor has not been appointed yet, and in the meantime the duties will be performed by the deputy medical superintendent.

Mr. Rooke Ley is to be congratulated on having obtained the largest pension ever granted to any county asylum medical superintendent. He is well known as one of the ablest of his confrères, and is deserving of everything he has received. At the same time we doubt whether retention of offices for such a long period is for the welfare either of the asylum or of the incumbent himself; and we are sure it is bad for all the junior members of the specialty. Promotion is checked just at the point where it is most important that it should flow easily, and for one who, like Mr. Ley, can hold his post satisfactorily after the age of sixty, there are a hundred who cannot. It is much to be desired that the Lunacy Acts Amendment Bill, which will soon make its annual appearance, will contain a clause of compulsory retirement of all Asylum Officials and of Commissioners in Lunacy on completion of sixty years of age. For the Lunacy Commission it is indeed more imperative than for asylums.

